# Prognostic value of modified criteria for hydroxyurea resistance or intolerance in patients with high‐risk essential thrombocythemia

**DOI:** 10.1002/cam4.5602

**Published:** 2023-01-09

**Authors:** Young Hoon Park, Sewon Lee, Yeung‐Chul Mun, Dong Jin Park

**Affiliations:** ^1^ Division of Hematology‐Oncology, Department of Internal Medicine Ewha Womans University Mokdong Hospital Seoul Republic of Korea; ^2^ Department of Laboratory Medicine Ewha Womans University Mokdong Hospital Seoul Republic of Korea

**Keywords:** essential thrombocythemia, hydroxyurea, intolerance, resistance

## Abstract

**Background:**

Recognizing intolerance or resistance to hydroxyurea (HU), which is related to the high risk of a disease transformation and reduced survival in essential thrombocythemia (ET), is crucial to making a reasonable decision about second‐line therapy. We assessed the prognostic impact of the modified European LeukemiaNet (mELN) criteria used in a recent MAJIC‐ET trial by analyzing the incidence of resistance or intolerance to HU and the survival outcome compared with those of the ELN criteria.

**Methods:**

We retrospectively compared the development of HU resistance or intolerance according to the ELN and mELN criteria for 148 high‐risk ET patients receiving HU between 2014 and 2018. The maximum tolerated dose for defining HU resistance was used in the mELN criteria.

**Results:**

The median age of patients was 65 years (range, 36–87), with a median follow‐up of 3.6 years (range, 1.1–6.4). Two thromboembolic events were observed during HU treatment. When applying the ELN criteria, 10 patients (6.9%) were resistant (*n* = 5 [3.4%]) or intolerant (*n* = 5 [3.4%]) to HU in comparison with 22 patients (15%, 14 [9.8%]) resistant and 8 [5.5%] intolerant when applying the mELN criteria. Transformation to myelofibrosis and acute myeloid leukemia occurred in 2 (1.4%) patients and 1 (0.7%) patient, respectively, as defined by the ELN criteria compared with 3 (2.1%) and 2 (1.4%) patients as defined by the mELN criteria. In multivariate analysis of transformation‐free survival, HU resistance defined by the mELN criteria but not the ELN criteria was an independent prognostic factor. In addition, HU resistance as defined by both sets of criteria was an independent risk factor for inferior overall survival. Intolerance of HU did not have any prognostic impact on survival.

**Conclusions:**

The mELN criteria are useful for identifying high‐risk ET patients who might be eligible for second‐line therapy in practice, which should be validated in a prospective setting.

## Graphical abstract

In multivariate analysis of OS, resistance to hydroxyurea according to the modified ELN or ELN criteria was independent prognostic factor. Resistance to hydroxyurea according to the modified ELN criteria was independent prognostic factors for TFS.

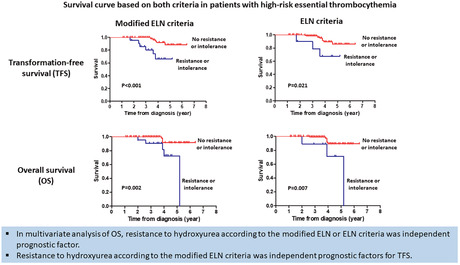

## 1 INTRODUCTION

Essential thrombocythemia (ET) is a classic Philadelphia‐negative myeloproliferative neoplasm associated with high risk of thrombotic/hemorrhagic complications and progression to myelofibrosis (MF) or acute myeloid leukemia (AML).^1^, ^2^, ^3^ The main treatment goal for patients with ET is to prevent thrombosis or bleeding events without increasing the risk of transformation, based on thrombosis risk.^4^, ^5^ According to the International Prognostic Score for Essential Thrombocythemia (IPSET)‐thrombosis model, the risk of thrombosis is estimated on the basis of age older than 60 years, a history of thrombosis, presence of a *JAK2V617F* mutation, and/or cardiovascular risk factors, and patients are categorized into four risk groups (very low, low, intermediate, and high).^1^, ^6^ Recently, the European LeukemiaNet (ELN) recommended cytoreduction therapy in high‐risk patients with ET (age >60 with *JAK2V617F* mutation or documented thrombosis history). Hydroxyurea (HU) with daily low‐dose aspirin is considered the frontline therapy for these patients, supported by data from four randomized studies.^6^, ^7^, ^8^, ^9^ However, approximately 20% of ET patients receiving cytoreduction therapy experience intolerance or resistance to HU, which is associated with high risk of disease transformation, inferior prognosis, and reduced overall survival.^2^, ^3^, ^10^ Recognizing intolerance or resistance to HU is important to make appropriate decisions about second‐line therapy to achieve disease control.

In 2007, the ELN suggested a unified definition of resistance and intolerance to HU for ET patients for use in scientific trials to establish endpoints for frontline therapy with HU or inclusion criteria for second‐line therapy after HU.^11^, ^12^ However, the definition was based on a consensus approach and was not supported by evidence‐based data; therefore, it is necessary to be validated in the clinical circumstances.^11^, ^13^ Recently, the MAJIC‐ET trial,^14^ a randomized, phase 2 study, evaluated the efficacy and safety of ruxolitinib compared with those of the best available treatment in ET patients who were resistant or intolerant to HU. In addition, a modification of the ELN criteria for HU resistance and intolerance was used. In this trial, the maximum tolerated dose (MTD) of HU and a minimum HU dose of 2000 mg/day in the ELN criteria were used for defining HU resistance. Unlike the ELN criteria, progressive hepatosplenomegaly or occurrence of thromboembolic/bleeding complications while on HU treatment was included as criteria for HU resistance. In addition, mucocutaneous symptoms, leg ulcers, HU‐related fever, and other unacceptable HU‐related side effects, which are frequently observed in clinical practice, were included as criteria for HU intolerance.

In this study, we aimed to assess the prognostic impact of the modified ELN (mELN) criteria for resistance and intolerance to HU in the MAJIC‐ET trial. We analyzed the incidence of resistance or intolerance to HU and the survival outcome according to both sets of ELN and mELN criteria.

## 2 MATERIALS AND METHODS

### 2.1 Patients and study design

From January 2010 to December 2020, a total of 357 patients (≥18 years) diagnosed with ET according to the World Health Organization (WHO) 2008 classification was screened at Ewha Womans University Mokdong Hospital. Among them, 165 patients who met the criteria for high‐risk ET based on the IPSET‐thrombosis model were included in this study. The high‐risk ET patients who had insufficient data regarding HU treatment (*n* = 12) and received other first‐line therapies such as anagrelide (*n* = 5) were excluded from this study. Finally, a total of 148 patients with high‐risk ET were analyzed retrospectively. The following clinical information was collected: age at diagnosis, sex, underlying comorbidities (especially, cardiovascular disease), complete blood count profile at diagnosis (white blood cells, hemoglobin, and platelets), lactate dehydrogenase (LDH) level, ET‐related microvascular symptoms, mutation status (*JAK2V617F*, *MPL*, *CALR*), presence of previous thrombosis event, the daily dose of HU, presence of disease transformation (AML or MF), and survival data. Toxicity data were assessed by the Common Terminology Criteria for Adverse Events (CTCAE) version 4.0. Based on the ELN guidelines, the criteria for response were defined as follows: (1) complete response (CR), platelet count ≤400 × 10^9^/L, no disease‐related symptoms, normal spleen size, and white blood cell count ≤10 × 10^9^/L; (2) partial response (PR), platelet count ≤600 × 10^9^/L or decrease >50% from baseline; and (3) no response (NR), any response that did not satisfy CR or PR. The appearance of HU resistance and intolerance was assessed by both ELN criteria and mELN criteria used in the MAJIC‐ET trial at any point in the disease course while on HU (Table 1). MTD is defined as the highest tolerated dose of HU that can be administered to patients with ET without causing moderate to severe toxicity (more than grade 2 toxicity). Depending on patients condition, HU usually was administered at a starting dose of 1000 mg/day and was increased until a minimum effective dose was achieved. If the patient experienced HU intolerance, a reduction of dose was allowed at the discretion of the attending physician. All patients received antiplatelet agents such as low‐dose aspirin or clopidogrel unless absolute contraindication for each drug was indicated. This study was approved by the Institutional Review Board of Ewha Womans University Mokdong Hospital and was performed in compliance with the Declaration of Helsinki.

**TABLE 1:** Definition of resistance or intolerance to hydroxyurea in patients with ET

ELN criteria	Modified ELN criteria
HU resistance	
(1) Platelet count >600 × 10^9^/L after 3 months of at least 2 g/day of HU (2.5 g/day in patients with a body weight >80 kg) (2) Platelet count >400 × 10^9^/L and WBC <2.5 × 10^9^/L at any dose of HU (3) Platelet count >4000 × 10^9^/L and hemoglobin less than 10 g/dL at any dose of HU	(1) Platelet count >600 × 10^9^/L after 8 weeks of at least 2 g/day or MTD of HU (2) Platelet count >400 × 10^9^/L and WBC <2.5 × 10^9^/L at any dose of HU (for a period of at least 8 weeks) (3) Platelet count >400 × 10^9^/L and hemoglobin <11 g/dL at any dose of HU (for a period of at least 8 weeks) (4) Platelet count persistently <100 × 10^9^/L at any dose of HU (for a period of at least 8 weeks) (5) Progressive splenomegaly or hepatomegaly (enlargement by more than 5 cm or appearance of new splenomegaly or hepatomegaly) on HU treatment (6) Not achieving the desired stable reduction of WBC when leukocytes are a target of therapy after 8 weeks of at least 2 g/day or MTD of HU (7) Thrombosis or hemorrhage (including transient ischemic attack) while on therapy
HU intolerance	
(1) Presence of leg ulcers or other unacceptable mucocutaneous manifestation at any dose of HU (2) HU‐related fever	(1) Presence of leg ulcers or other unacceptable HU‐related nonhematological toxicities, such as unacceptable mucocutaneous manifestations, gastrointestinal symptoms, pneumonitis, or fever at any dose of HU. (2) Disease‐related symptoms not controlled by HU

Abbreviations: ET, essential thrombocythemia; ELN, European LeukemiaNet; HU, hydroxyurea; MTD, maximum tolerated dose; WBC, white blood cell.

### 2.2 Statistical analysis

Continuous and categorical variables are summarized as median, mean, or number of patients and percentage, respectively. Overall survival (OS) was defined as the time interval between the date of diagnosis and the date of death by any cause. Transformation‐free survival (TFS) was estimated from the date of diagnosis to the date of disease transformation. Kaplan–Meier analysis was used to estimate cumulative survivals, and the differences between survival curves were analyzed using the log‐rank test. All *p* values presented were two‐sided and *p* < 0.05 was considered statistically significant. All statistical analyses were performed using SPSS version 24.0 (IBM.).

## 3 RESULTS

### 3.1 Baseline characteristics

Patient and disease characteristics at diagnosis are summarized in Table 2. The median age was 65 years (range, 36–87) at the time of diagnosis, and 90 (60.8%) patients were male. Among all patients, 121 (81.8%) were older than 60 years. Eighteen patients (12.2%) had a history of thrombosis before being diagnosed with ET. Of them, myocardial infarction (*n* = 5) and deep vein thrombosis (*n* = 5) were the most common thrombotic events in arterial and venous sites, respectively. Two patients had a history of bleeding (intracranial hemorrhage and colon diverticular bleeding). Most patients had at least one cardiovascular risk factor such as hypertension (37.2%), diabetes mellitus (16.9%), or dyslipidemia (33.1%). Forty‐four patients received antiplatelet agents such as aspirin (*n* = 32), clopidogrel (*n* = 8), or both (*n* = 4) at diagnosis. The median platelet count was 880 × 10^9^/L (range, 480–1500), and hepatosplenomegaly on physical examination or imaging was present in 12.8% of patients. Regarding mutation status, 72 patients (48.6%) harbored a *JAK2V617F* mutation. *MPL* and *CALR* mutations were found in 19.6% and 12.8% of patients, respectively.

**TABLE 2:** Baseline characteristics of high‐risk ET patients treated with hydroxyurea

Characteristics	*N* = 148
Age at diagnosis, median (range), years	68 (46–87)
Age >60 years, *n* (%)	121 (81.8)
Male/female, *n* (%)	58 (39.1)/90 (60.8)
Cardiovascular risk, *n* (%)	
Hypertension	55 (37.2)
Diabetes mellitus	25 (16.9)
Dyslipidemia	49 (33.1)
Others	8 (5.4)
History of thrombosis events, *n* (%)	18 (12.2)
Arterial events	10 (6.8)
Venous events	8 (5.4)
History of any bleeding events, *n* (%)	2 (1.4)
Antiplatelet medication, *n* (%)	
Aspirin	32 (21.6)
Clopidogrel	8 (5.4)
Both aspirin and clopidogrel	4 (2.7)
CBC profiles at diagnosis, median (range)	
WBC count (×10^9^/L)	8.8 (4.5–23)
Hemoglobin (g/dL)	14.8 (8.3–18.5)
Platelet count (×10^9^/L)	880 (480–1500)
High LDH level, *n* (%)	102 (68.9
Hepatosplenomegaly, *n* (%)	19 (12.8)
Microvascular symptoms, *n* (%)	69 (46.6)
Mutation status, *n* (%)	
*JAK2V617F* mutation	72 (48.6)
*MPL* mutation	29 (19.6)
*CALR* mutation	19 (12.8)
Triple negative	5 (3.4)
Missing	21 (14.2)
Daily dose of hydroxyurea, mg/day	
Mean/median/range	1142/1000/500–2500
>2 g/day	1 (0.7)

Abbreviations: CBC, complete blood count; WBC, white blood cell; LDH, lactate dehydrogenase.

### 3.2 HU treatment outcomes

The median daily dose of HU was 1000 mg (range, 500–2000 mg), and only one patient received more than 2000 mg/day for at least 8 weeks. HU‐related adverse effects occurred in 30.4% of patients and generally were grades 1 and 2 and developed within 5 months after HU treatment. Intermittent mild cytopenia (anemia and leukopenia) and mucocutaneous hyperpigmentation were observed frequently, sometimes leading to dose reduction or temporary discontinuation. Regarding treatment response, the best response during the course of HU treatment was CR in 85 patients (55.9%), PR in 56 patients (39.2%), and NR in 7 patients (4.9%). No HU‐related death or secondary nonhematologic malignancy was observed.

### 3.3 HU resistance

Overall, resistance to HU was shown in 5 (3.5%) and 14 (9.8%) patients when using the ELN and the mELN criteria, respectively. One patient received 2500 mg/day of HU for 3 months, which was defined as HU resistance by both sets of criteria. Seven patients had a platelet count >600 × 0^9^/L after 3 months of therapy at diverse HU doses, ranging from 1500 to 2000 mg/day (median 1500). Even though insufficient platelet response was achieved during treatment, no further HU dose escalation (more than 2000 mg/day) was performed due to the presence of HU‐related adverse effects and physician concern for the occurrence of such toxicities, which was defined as resistance to HU by the MTD concept used in the mELN criteria. Three (2.1%) patients met the criteria of resistance due to anemia (hemoglobin <10 g/dL) with a platelet count >400 × 10^9^/L at an HU dose of 1000 mg (*n* = 2) or 1500 mg (*n* = 1). Leukopenia (2.2 × 10^9^/L) with a platelet count >400 × 10^9^/L occurred in one patient at a dose of 1500 mg/day. This patient stopped HU treatment and received only antiplatelet therapy. No patients displayed progressive hepatosplenomegaly. A total of two thromboembolic events were observed during HU treatment (transient ischemic attack and deep vein thrombosis), which is categorized as resistance as defined by the mELN but not the ELN criteria. No bleeding events occurred during HU therapy. HU‐resistant patients defined by the ELN criteria or the mELN criteria switched to either a reduced dose of HU combined with anagrelide therapy (*n* = 3 vs. *n* = 9) or anagrelide monotherapy (*n* = 2 vs. *n* = 5).

### 3.4 HU intolerance

HU intolerance was found in 5 (3.4%) and 8 (5.6%) patients when applying the ELN and the mELN criteria, respectively (Table 3). Mucocutaneous manifestations (hyperpigmentation, aphthous ulcer, and mucositis) were the most common HU‐related toxicities and developed in 5 (3.4%) patients. Two (1.4%) patients had grade 3 HU‐related nausea/vomiting at a dose of 1500 mg/day, which resolved after HU discontinuation. ET‐related persistent fatigue was not improved in one patient even though the daily HU dose was increased from 1000 mg to 2000 mg. No leg ulcers or HU‐related fever was observed during the study period. All HU‐intolerant patients switched from HU therapy to either reduced HU dose plus anagrelide combination therapy or anagrelide monotherapy, resulting in favorable responses and tolerable safety profiles.

**TABLE 3:** Occurrence of HU resistance and intolerance: The ELN versus the modified ELN criteria

	ELN (*n* = 148)	Modified ELN (*n* = 148)
Resistance or intolerance, *n* (%)	10 (6.9)	22 (15)
Resistance, *n* (%)	5 (3.4)	14 (9.8)
PLT > 600 × 10^9^/L after 8 weeks of at least 2 g/day or MTD of HU	1 (0.7)	8 (5.6)
PLT > 400 × 10^9^/L and WBC <2.5 × 10^9^/L at any dose of HU^a^	1 (0.7)	1 (0.7)
PLT > 400 × 10^9^/L and hemoglobin <11 g/dL at any dose of HU^a^	3 (2.1)	3 (2.1)
PLT persistently <100 × 10^9^/L at any dose of HU^a^	—	0 (0)
Progressive splenomegaly or hepatomegaly	—	0 (0)
Thrombosis or hemorrhage (including TIA) while on therapy	—	2 (1.4)
Intolerance, *n* (%)	5 (3.4)	8 (5.6)
Unacceptable mucocutaneous manifestations	5 (3.4)	5 (3.4)
HU‐related fever	0 (0)	0 (0)
Other unacceptable nonhematological toxicities^b^	—	2 (1.4)
Disease‐related symptoms not controlled by HU	—	1 (0.7)

Abbreviations: HU, hydroxyurea; ELN, European LeukemiaNet; PLT, platelet; MTD, maximum tolerated dose; WBC, white blood cell; TIA, transient ischemic attack.

^a^ For a period of at least 8 weeks.

^b^ Such as unacceptable gastrointestinal symptoms or pneumonitis at any dose of hydroxyurea, except fever.

### 3.5 Transformation and overall survival

Overall, at a median follow‐up of 3.6 years (range, 1.1–6.4), transformation to MF and AML occurred in 8 (5.5%) and 4 (2.8%) patients, respectively. Of them, 7 died of disease transformation. Transformation to MF or AML was seen in three patients and one patient in the HU‐resistant group as defined by the mELN criteria. In HU‐resistant patients defined by the ELN criteria, two patients experienced disease transformation to MF (*n* = 1) or AML (*n* = 1). There was only one disease transformation (1 MF) in HU‐intolerant patients as defined by both sets of criteria.

**FIGURE 1:**
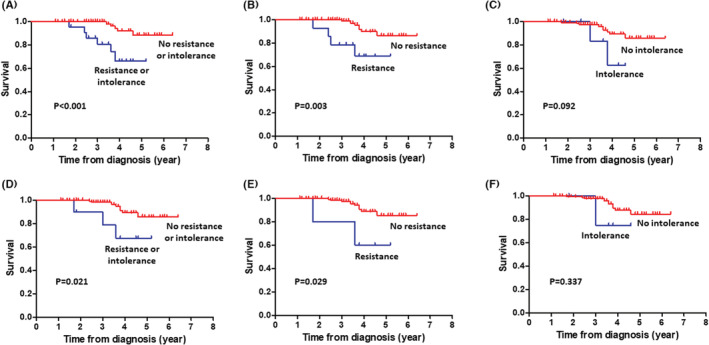
Transformation‐free survival. Patients with either resistance or intolerance (A), resistance (B), and intolerance (C) to hydroxyurea as defined by the modified ELN criteria. Patients with either resistance or intolerance (D), resistance (E), and intolerance (F) to hydroxyurea as defined by the ELN criteria. ELN, European LeukemiaNet

In terms of TFS, HU‐resistant or intolerant patients showed reduced TFS compared with those who had no resistance or intolerance to HU when using the mELN (*p* < 0.001) and the ELN (*p* = 0.021) criteria (Figure 1A,D). Univariate analysis showed that TFS was significantly different between HU‐resistant patients and nonresistant patients despite the criteria applied (hazard ratio [HR] = 3.83, 95% confidence interval [CI], 1.754–8.569; *p* = 0.003) in the mELN criteria and HR = 2.19, 95% CI 1.071–4.505, *p* = 0.029 in the ELN criteria (Figure 1B,C, Table 4). No statistically significant association was observed between HU‐intolerance and TFS when applying either ELN or mELN criteria (*p* = 0.337 and *p* = 0.092, respectively) (Figure 1C,F). In addition, univariate analysis of TFS showed that older age (>70 years, HR = 2.52, 95% CI 1.160–5.501, *p* = 0.012), presence of anemia (Hb <12 g/dL, HR = 2.92, 95% CI 1.248–12.233, *p* = 0.019), prior thrombosis history (HR = 4.25, 95% CI 2.506–24.785, *p* = 0.015), leukocytosis (>10 × 10^9^/L, HR = 1.82, 95% CI 1.105–3.568, *p* = 0.041), and high LDH levels (HR = 1.68, 95% CI 1.114–2.425, *p* = 0.025) were associated with worse TFS in the present cohort. In multivariate analysis of TFS, older age (>70 years, HR = 6.81, 95% CI 2.668–17.528, *p* < 0.001), presence of anemia (Hb <12 g/dL, HR = 5.55, 95% CI 1.745–17.655, *p* < 0.001), and resistance to HU according to the mELN criteria (HR = 3.825, 95% CI 1.687–8.851, *p* = 0.001) were independent prognostic factors (Table 4).

**TABLE 4:** Univariate and multivariate analyses of transformation‐free survival and overall survival

Variables	Transformation‐free survival				Overall survival			
	Univariate analysis		Multivariate analysis		Univariate analysis		Multivariate analysis	
	HR (95% CI)	*p*‐value	HR (95% CI)	*p*‐value	HR (95% CI)	*p*‐value	HR (95% CI)	*p*‐value
Age >70 years	2.52 (1.160–5.501)	0.012	6.81 (2.668–17.528)	<0.001	3.90 (2.244–13.571)	<0.001	4.85 (2.347–15.471)	<0.001
Female	0.74 (0.383–1.488)	0.398	—	—	1.34 (0.477–3.698)	0.128	—	—
Prior thrombosis history	4.25 (2.506–24.785)	0.015	2.58 (0.782–5.422)	0.528	4.85 (2.561–27.884)	<0.001	5.66 (2.784–29.456)	0.002
Hemoglobin <12 g/dL	2.92 (1.248–12.233)	0.019	5.55 (1.745–17.655)	<0.001	2.36 (1.651–11.584)	0.028	2.18 (0.777–8.544)	0.128
WBC count >10 × 10^9^/L	1.82 (1.105–3.568)	0.041	2.25 (0.958–6.874)	0.258	1.65 (1.215–2.368)	0.665	—	—
PLT count >1000 × 10^9^/L	1.86 (0.986–3.668)	0.122	—	—	1.58 (0.387–2.854)	0.583	—	—
High LDH level	1.68 (1.114–2.425)	0.025	2.86 (0.942–8.755)	0.125	1.92 (1.993–16.232)	0.019	2.57 (0.257–4.588)	0.295
Splenomegaly	1.28 (0.058–1.645)	0.587	—	—	1.45 (0.658–3.699)	0.551	—	—
JAK2 mutation	1.26 (1.115–2.253)	0.685	—	—	1.82 (0.699–5.625)	0.923	—	—
Microvascular symptom	1.32 (0.664–3.325)	0.375	—	—	1.28 (0.687–3.688)	0.160	—	—
Cardiovascular risk	1.15 (0.378–1.482)	0.254	—	—	1.22 (0.348–2.641)	0.924	—	—
Resistance by the ELN	2.19 (1.071–4.505)	0.029	3.977 (0.954–7.576)	0.056	2.12 (2.689–12.677)	0.025	3.98 (2.743–16.941)	0.001
Resistance by the mELN	3.83 (1.754–8.569)	0.003	3.825 (1.687–8.851)	0.001	2.89 (2.789–18.124)	0.015	4.11 (2.966–20.477)	<0.001
Intolerance by the ELN	1.25 (0.564–3.258)	0.337	—	—	1.68 (0.388–2.587)	0.156	—	—
Intolerance by the mELN	1.98 (0.743–2.228)	0.092	—	—	1.68 (0.388–2.587)	0.078	—	—

Abbreviations: CI, confidence interval; ELN, European LeukemiaNet; HR, hazard ratio; LDH, lactate dehydrogenase; mELN, modified European LeukemiaNet; PLT, platelet; WBC, white blood cell.

**FIGURE 2:**
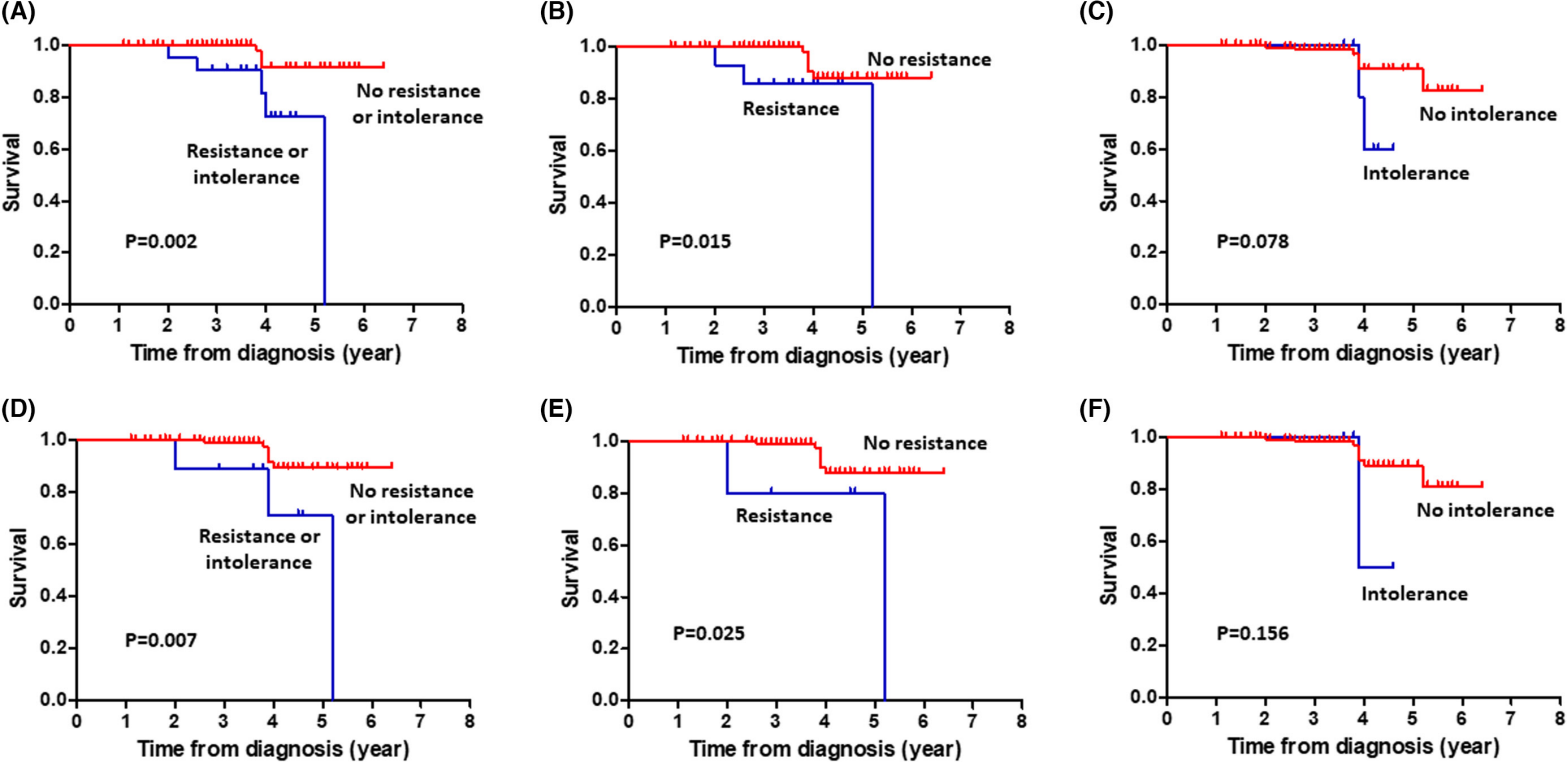
Overall survival. Patients with either resistance or intolerance (A), resistance (B), and intolerance (C) to hydroxyurea as defined by the modified ELN criteria. Patients with either resistance or intolerance (D), resistance (E), and intolerance (F) to hydroxyurea as defined by the ELN criteria. ELN, European LeukemiaNet

In terms of OS, HU‐resistant/intolerant patients displayed an inferior OS compared with those who had no resistance/intolerance to HU when applying either the mELN (*p* = 0.008) or the ELN (*p* = 0.021) criteria (Figure 2A,D). Univariate analysis of OS showed that HU resistance was significantly associated with inferior OS by either mELN (HR = 2.89, 95% CI 2.789–18.124, *p* = 0.015) or ELN (HR = 2.12, 95% CI 2.689–12.677, *p* = 0.025) criteria (Figure 2B,E). However, intolerance to HU did not show any prognostic impact on OS for either of the two sets of criteria that were applied (Figure 2C,F). In addition, univariate analysis of OS showed that older age (>70 years, HR = 3.90, 95% CI 2.244–13.571, *p* < 0.001), presence of anemia (Hb <12 g/dL, HR = 2.36, 95% CI 1.651–11.584, *p* = 0.028), prior thrombosis history (HR = 4.85, 95% CI 2.561–27.884, *p* < 0.001), and high LDH levels (HR = 1.92, 95% CI 1.993–16.232, *p* = 0.019) were associated with worse OS in the present cohort. In multivariate analysis of OS, older age (>70 years, HR = 4.85, 95% CI 2.347–15.471, *p* < 0.001), prior thrombosis history (HR = 5.66, 95% CI 2.784–29.456, *p* = 0.002), and resistance to HU according to mELN (HR = 4.11, 95% CI 2.933–20.477, *p* < 0.001) or ELN (HR = 3.98, 95% CI 2.743–16.941, *p* = 0.001) criteria were independent prognostic factors (Table 4).

## 4 DISCUSSION

We evaluated criteria for resistance or intolerance to HU, which are used widely in cytoreduction therapy for high‐risk ET cohorts. This study showed that the mELN criteria had prognostic value in predicting TFS and OS, like the ELN criteria. Due to the clinical importance of recognizing intolerance or resistance to HU, which is related to disease progression and inferior survival, ELN experts proposed standardized criteria for evaluating clinically significant resistance and intolerance to HU in ET to support clinical decision‐making.^12^ These criteria have been evaluated in clinical trials for ET, having gained general acceptance in both research studies and clinical practice.^13^ Previous studies showed that about 10% of patients with ET were intolerant or resistant to HU when the ELN criteria were applied.^1^, ^2^, ^4^, ^13^ In the present study, 6.9% of patients were classified as HU resistant or intolerant, a relatively low incidence compared with levels reported in previous studies.^1^, ^2^, ^4^, ^13^ It is probable that the short median time of follow‐up played a role in this result because resistance to HU was infrequent and usually occurred late during the course of ET.

In routine practice, it is not always easy to achieve adequate platelet response at high HU doses. Many physicians tend not to prescribe high‐dose HU due to concern over HU‐related toxicities, which are associated with the dose. In several reported studies, the median daily dose of HU usually did not exceed 2000 mg, which is similar to the average dose in our cohort.^3^, ^7^, ^13^, ^15^, ^16^, ^17^ In our study, only one patient received more than 2000 mg/day of HU for at least 3 months, finally failing to achieve a platelet count <600 × 10^9^/L and was defined as HU resistant based on the ELN criteria. Seven patients had insufficient platelet response at HU doses less than 2000 mg/day, which was defined as resistance to HU only by the mELN criteria. By introducing the concept of MTD, even if physicians did not use high‐dose HU (>2000 mg/day) to achieve a better balance between disease control and toxicity, the criteria for resistance were broadened, allowing the identification of patients with HU resistance who might benefit from secondary therapeutic options. Because high‐dose HU administration does not seem to be common, it might be more appropriate for daily clinical practice to use the mELN resistance criteria. In addition, persistent thrombocytopenia (<100 × 10^9^/L) at any dose of HU could be a meaningful indicator of resistance because it could reflect better a lack of hematopoietic reserve and near disease transformation than a dose‐dependent process, like anemia and leukopenia, which were included in the ELN resistance criteria. As the concept of HU resistance expands, the new mELN criteria can help physicians more accurately identify patients who need second‐line therapy for ET in real‐world practice. However, further studies in large populations aimed at validating these findings are warranted.

Previously, it was shown that anemia, old age, previous thrombosis, and extreme thrombocytosis (platelet ≥1000 × 10^9^/L) had prognostic significance relative to existing risk factors for disease transformation and inferior OS in ET.^1^, ^2^, ^3^, ^13^, ^18^ In our study, increased age (>70 years), hemoglobin level below normal, and HU resistance as defined by the mELN criteria were independent risk factors for poor TFS. Regarding OS, old age, prior thrombosis history, and HU resistance as defined by both the ELN and mELN criteria were independent risk factors for inferior OS. These results are similar to those of previously reported studies.^1^, ^2^, ^13^ However, in regard to TFS, resistance as defined by the ELN criteria did not maintain prognostic significance during multivariate analysis, which resulted from the relatively small number of patients and the short follow‐up time in our study. We demonstrated that resistance to HU was a significant adverse prognostic factor for survival and hematologic transformation, requiring confirmation in a prospective setting. In addition, similar to the results of previous studies, HU intolerance defined by both sets of criteria was not identified as an independent predictor of inferior survival.

In our study, HU intolerance was observed in 3.4% (by ELN criteria) and 5.6% (by mELN criteria) of patients, respectively, which is slightly lower than that reported in previous studies.^1^, ^2^, ^4^, ^10^, ^11^, ^13^, ^14^, ^19^ As the criteria for intolerance expanded, the incidence rate of HU intolerance was higher in the mELN than in the ELN. In clinical practice, some patient subgroups experienced severe HU‐related adverse effects other than mucocutaneous symptoms, including gastrointestinal symptoms, loss of appetite, or weight gain, following discontinuation or dose reduction of HU and initiation of second‐line therapy. In addition, ET‐related symptoms not controlled by HU dose modification, which significantly impaired the quality of life, were found in some ET patients. In our study, one patient experienced intractable fatigue due to ET during HU therapy and achieved tolerable symptom relief after changing to second‐line therapy. However, the current ELN criteria do not include these symptom categories as intolerance. Even though intolerance to HU does not entail prognostic significance in survival outcomes when the two sets of criteria were applied in our study, intolerance due to severe side effects or high disease burden of ET lead to the reduction of HU and poor compliance to the first‐line HU therapy, potentially interfering with treatment efficacy. This is thought to be an important and relevant clinical factor that should be included in the criteria for intolerance. Applying the new definition of intolerance, which was included in the mELN criteria in this study, early application of second‐line therapy can be beneficial for the patients who failed first‐line therapy with unacceptable symptom burden.

Resistance to HU appears to lead to an increased risk of transformation to MF and AML and death, emphasizing the importance of second‐line therapeutic options for these patients. Currently, however, there are no definite guidelines on how to manage patients with high‐risk ET who develop resistance or intolerance to HU. For these patients, the ELN has recommended anagrelide, an inhibitor of megakaryocyte differentiation and proliferation, as a second‐line therapy^8^, ^20^ Some small studies suggest a combination of low‐dose HU and anagrelide, which can be useful in controlling thrombocytosis with fewer adverse effects for HU‐intolerant ET patients.^15^, ^16^ In this study, most HU‐resistant patients received lower dose HU plus anagrelide combination therapy after HU monotherapy at higher doses, which was better tolerated and led to better control of thrombocytosis. Pegylated‐interferon (peg‐IFN) showed outstanding efficacy in achieving cytoreduction and molecular response in patients with ET, suggesting that peg‐IFN could be effective in HU‐resistant or intolerant patients with ET as second‐line therapy.^21^, ^22^ Unlike a prospective trial of patients with polycythemia vera, ruxolitinib, a *JAK2* inhibitor, did not show better efficacy than the best available therapy in HU‐resistant or ‐intolerant patients with ET in the MAJIC‐ET trial.^14^

Our study is limited by its retrospective nature, which might limit the interpretation of the results. Adverse events could be underestimated due to the retrospective nature of the study which depended mainly on medical records offered by attending physicians, not by uniform documents. In addition, the relatively small number of patients and short follow‐up period might yield unavoidable selection bias. Even if these limitations undermine the validity and reliability of the conclusions, the actual data from this study are helpful for future research.

In conclusion, HU intolerance or resistance, as defined by mELN criteria, is useful for identifying high‐risk ET patients who might be eligible for salvage therapy. However, the value of the mELN criteria should be validated in a prospective setting with large population size.

## AUTHOR CONTRIBUTIONS

**Young Hoon Park:** Data curation (lead); formal analysis (equal); investigation (equal); methodology (equal); resources (equal); writing – original draft (lead); writing – review and editing (equal). **Sewon Lee:** Data curation (equal); investigation (equal); resources (equal); writing – review and editing (equal). **Yeung‐Chul Mun:** Data curation (equal); investigation (equal); methodology (equal); writing – review and editing (equal). **Dong Jin Park:** Conceptualization (equal); data curation (equal); investigation (equal); methodology (equal); supervision (equal); validation (equal); writing – original draft (equal); writing – review and editing (equal).

## FUNDING INFORMATION

No specific funding was disclosed.

## CONFLICT OF INTEREST

The authors made no disclosures.

## ETHICS STATEMENT

This study was approved by the Institutional Review Board of Ewha Womans University Mokdong Hospital with a waiver of informed consent owing to its retrospective nature (IRB No. EUMC 2021‐03‐035) and was performed in compliance with the Declaration of Helsinki.

## Data Availability

Data sets used and analyzed in this study are available from the corresponding author upon reasonable request.
